# The Role of Psychosocial Stressors in Shaping the Attitudes of Nurses in a Canadian Acute Care Hospital Toward Patients Who Use Substances: A Cross‐Sectional Study

**DOI:** 10.1155/jonm/6956980

**Published:** 2026-08-27

**Authors:** Andrea Raynak, Michel Bédard, Brianne Wood, Christopher Mushquash

**Affiliations:** ^1^ Nursing Practice & Learning, Thunder Bay Regional Health Sciences Centre, Thunder Bay, Ontario, Canada, tbrhsc.net; ^2^ Department of Health Sciences, Lakehead University, Thunder Bay, Ontario, Canada, lakeheadu.ca; ^3^ Centre for Applied Health Research, St. Joseph’s Care Group, Thunder Bay, Ontario, Canada; ^4^ Thunder Bay Regional Health Sciences Centre, Centre for Social Accountability, NOSM University, Thunder Bay, Ontario, Canada, tbrhsc.net; ^5^ Canada Research Chair in Indigenous Mental Health and Addiction, Thunder Bay, Ontario, Canada; ^6^ Department of Psychology, Lakehead University, Thunder Bay, Ontario, Canada, lakeheadu.ca; ^7^ Northern Ontario School of Medicine University, Thunder Bay, Ontario, Canada; ^8^ Dilico Anishinabek Family Care, Thunder Bay, Ontario, Canada; ^9^ Thunder Bay Regional Health Sciences Centre, Thunder Bay, Ontario, Canada, tbrhsc.net; ^10^ Thunder Bay Regional Health Research Institute, Thunder Bay, Ontario, Canada, tbrhri.ca

**Keywords:** attitude of health personnel, burnout, nurses, professional, social desirability, substance-related disorders, workload

## Abstract

**Aim:**

To examine the extent to which psychosocial stressors, including social desirability, workload, burnout, and adverse childhood experiences (ACEs), were associated with nurses’ attitudes toward patients who use substances in hospital settings.

**Design:**

A cross‐sectional survey design was used.

**Methods:**

The study was conducted in a 425‐bed tertiary care hospital in Northwestern Ontario, Canada. Registered nurses and registered practical nurses providing direct inpatient care were eligible to participate. Data were collected between October 14 and November 4, 2024, via an electronic survey distributed through institutional email using REDCap. A total of 412 participants completed validated instruments measuring attitudes toward patients who use substances, social desirability, workload, burnout, and ACEs. Hierarchical multiple regression analyses were used to examine associations between these variables and nurses’ attitudes.

**Results:**

Professional development in substance use and addiction emerged as the most consistent variable associated with more positive attitudes, demonstrating a dose–response relationship; nurses reporting ≥ 9 h of training had significantly more favorable attitudes compared with those with no training. Social desirability was significantly associated with attitudes, suggesting potential underreporting of negative views. Workload and burnout demonstrated variable, scale‐dependent associations, while ACEs were not significantly associated with attitudes. Nurses working in inpatient mental health settings reported more positive attitudes than those in other clinical areas.

**Conclusion:**

Professional development, particularly of greater duration and intensity, was identified as a key modifiable factor associated with more positive attitudes toward patients who use substances. These findings highlight actionable opportunities for nursing leadership to strengthen practice environments through sustained education, attention to psychosocial workplace factors, and context‐specific supports. Integrating these strategies into workforce planning may enhance nurse well‐being and contribute to more equitable, stigma‐reducing care in hospital settings.

## 1. Introduction

In Canada, substance use and substance‐use disorders remain a significant public health concern, with rising rates of opioid‐related harms and hospitalizations across multiple provinces [1, 2]. Nurses represent the largest group of healthcare providers within the Canadian health system and are central to the delivery of hospital‐based care [3]. Entry‐to‐practice nursing education in Canada emphasizes mental health and addiction competencies, including the provision and evaluation of person‐centered care in partnership with individuals experiencing substance use across the continuum of care and lifespan [4]. These competencies extend to acute care nurses, who frequently serve as the primary point of contact and play a central role in pain management, withdrawal monitoring, and care coordination [5]. As such, their attitudes can directly influence care experiences, patient engagement, and clinical outcomes.

Yet, researchers report that nurses hold less favorable attitudes toward patients who use substances [6–8]. Nurses have described feeling dissatisfied and disengaged when caring for this population [9–11], as well as challenged by behaviors they perceive as disruptive or difficult [12, 13]. Some have also expressed hesitation or discomfort in providing care [14]. These experiences have been associated with heightened emotional exhaustion and distress [8–10], and attitudes toward these patients have been shown to be more negative compared with those toward patients with other comorbidities [15].

Nurses’ judgmental attitudes may contribute to stigmatizing care experiences for patients who use substances. Patients have reported feeling stigmatized in hospital settings, often being perceived as uncooperative, difficult, or undeserving of care [16, 17]. Patients have described experiences of discrimination, including being ignored by nurses [18], being denied pain medication [19, 20], and having their concerns dismissed [21, 22]. These experiences contribute to elevated rates of patients leaving hospital before completing treatment [22, 23].

Nurses have identified several factors shaping these attitudes, including (a) limited organizational support, particularly the absence of clear policies and procedures to guide care [8, 12, 24]; (b) insufficient knowledge related to substance use and addiction [8, 10, 11, 25]; and (c) challenges in pain management, often framed through concerns related to substance use and the assessment and management of analgesia in patients with substance‐use histories [8, 12]. Despite this, less attention has been paid to broader psychosocial factors that may also shape these attitudes.

Such factors may include workload and burnout, both of which have intensified in the postpandemic context and may impair emotional resilience and contribute to job dissatisfaction [26, 27], potentially exacerbating negative attitudes toward already stigmatized populations. Similarly, although personal experience with substance use within one’s social network has been associated with more positive attitudes [7, 10, 11], the influence of adverse childhood experiences (ACE) (e.g., abuse, neglect, or family dysfunction) on nurses’ perceptions has not been examined. Societal expectations and professional norms may encourage socially desirable responding rather than authentic expression, suggesting a potential role for social desirability bias in attitudinal research. Collectively, these factors offer a more comprehensive lens through which to understand the influences shaping nurses’ attitudes in hospital settings. A nuanced understanding of these psychosocial influences can equip nursing administrators to make evidence‐informed decisions that better support nurses while promoting safe, equitable, and patient‐centered care. Accordingly, the purpose of this study was to examine the extent to which workload, burnout, ACEs, and social desirability were associated with nurses’ attitudes toward patients who use substances in hospital settings using hierarchical regression analyses.

## 2. Methods

### 2.1. Study Design

This cross‐sectional study was reported in accordance with the Strengthening the Reporting of Observational Studies in Epidemiology (STROBE) guidelines [28]. An electronic survey was used to collect data for this study. The survey was distributed to registered nurses (RNs) and registered practical nurses (RPNs) working in a teaching hospital in northwestern Ontario, Canada. Research ethics board approval was obtained on October 3, 2024 (#1469703). The study adhered to the ethical principles outlined in the Declaration of Helsinki and the Tri‐Council Policy Statement: Ethical Conduct for Research Involving Humans.

The survey was distributed via email by the professional practice department to all 1400 RNs and RPNs employed at the hospital on October 14, 2024. A reminder email was sent on October 28, 2024. The survey remained open for 3 weeks, closing on November 4, 2024. Recruitment posters containing a QR code and study information were displayed throughout the hospital and shared on the internal electronic communication board. Light refreshments were provided during in‐person recruitment sessions; however, no monetary incentives were offered for survey completion.

### 2.2. Study Sample

Convenience sampling was used to obtain the study sample. Eligible participants included RNs and RPNs with current registration in Ontario, Canada, who were employed in casual, part‐time, full‐time, or temporary positions and provided direct patient care. Nurses in nondirect care roles, including nurse practitioners, clinical nurse specialists, and those in leadership positions (e.g., manager, coordinator, and director), were excluded, as these roles do not typically involve ongoing patient contact or sustained therapeutic relationships with patients.

### 2.3. Study Setting

The study was conducted at a teaching hospital that serves as the primary acute care and tertiary referral center. The hospital operates a 425‐bed facility and provides a broad range of specialized services. It serves a catchment population of over 250,000 individuals across a large geographic region encompassing urban, rural, and remote communities.

### 2.4. Study Measures

#### 2.4.1. Instruments of Measurement: Dependent Variables

Participants were randomized to complete either the original AAPPQ/DDPPQ instruments [29, 30] or the person‐centered wording adaptations developed by Mahmoud et al. [10, 11] (PC‐AAPPQ/Person‐Centered DDPPQ [PC‐DDPPQ]). However, in a prior psychometric analysis conducted using the current dataset [31], the previously published factor structures for the person‐centered versions were not supported. Consequently, revised versions with fewer items and different factor structures were empirically derived and subsequently used for the analyses reported in this manuscript.

##### 2.4.1.1. AAPPQ and PC‐AAPPQ

The AAPPQ was originally developed as a multidimensional instrument [32]. Subsequent psychometric evaluations have supported a 30‐item, six‐factor structure, including the role support domain (Gorman and Cartwright, 1991), with reported Cronbach’s alpha coefficients ranging from 0.66 to 0.85. Mahmoud et al. [33] developed the Person‐Centered AAPPQ (PC‐AAPPQ), which retains the original item content, structure, scoring, and interpretation while replacing stigmatizing terminology (e.g., “drinker”) with neutral, person‐centered language (e.g., “individuals who drink alcohol”). For both versions, items are rated on a 5‐point Likert‐type scale ranging from 1 (*strongly disagree*) to 5 (*strongly agree*), with higher scores reflecting more negative attitudes toward individuals who use alcohol. Psychometric evaluation of the PC‐AAPPQ demonstrated acceptable model fit (CFI = 0.871, SRMR = 0.071, and RMSEA = 0.06), with internal consistency reliability for the subscales ranging from 0.609 to 0.917 [33].

The revised version of the PC‐AAPPQ reported by Raynak and Colleagues [31], and used for the analyses presented in the following, consists of 22 items and a four‐factor structure. This factor structure, as determined by exploratory factor analysis, included the initial removal of items 9, 11, 15, 17, and 20. Further interitem correlation analysis led to the removal of Items 10, 18, and 19, yielding the final 22‐item model. The factors and items are as follows: role adequacy (Items 1–5), confidence, role clarity and support (Items 6–8, and 12–14), work satisfaction and motivation (Items 16 and 21–26), and self‐efficacy and emotional satisfaction (Items 27–30). The rating and scoring of the items remain unchanged (i.e., the same five‐point Likert scale with higher ratings indicating more negative attitudes). The four factors are (1) role adequacy, (2) confidence, role clarity, and support, (3) work satisfaction and motivation, and (4) self‐efficacy and emotional satisfaction.

##### 2.4.1.2. DDPPQ and PC‐DDPPQ

The DDPPQ was adapted by Watson et al. [30] from the AAPPQ [32] to assess health professionals’ attitudes toward working with individuals who use drugs. Subsequent psychometric evaluation supported a 20‐item, five‐factor structure comprising role support, role legitimacy, role adequacy, role‐related self‐esteem, and job satisfaction [30], with reported Cronbach’s alpha coefficients ranging from 0.78 to 0.86. Mahmoud et al. [11] developed the PC‐DDPPQ, which retains the original item content, structure, scoring, and interpretation while replacing stigmatizing terminology (e.g., “drug user”) with neutral, person‐centered language (e.g., “individuals who use drugs”). For both versions, items are rated on a 5‐point Likert‐type scale ranging from 1 (*strongly disagree*) to 5 (*strongly agree*), with higher scores reflecting more negative attitudes toward individuals who use drugs. Psychometric evaluation of the PC‐DDPPQ supported a 19‐item, five‐factor structure and demonstrated good model fit (CFI = 0.959, TLI = 0.951, and RMSEA = 0.058), with Cronbach’s alpha coefficients ranging from 0.725 to 0.937 [11].

The revised version of the PC‐DDPPQ reported by Raynak et al. [31], and used for the analyses presented in the following, consists of 16‐items and a 4‐factor structure. This factor structure, as determined with exploratory factor analysis, led to the initial removal of items 8, 9, and 13. Interitem correlation analysis led to the removal of Item 14, resulting in a 16‐item scale. The factors and items are as follows: role adequacy (Items 1–7), role support (Items 10–12), perceived competence and emotional response (Items 15–17), and job satisfaction (Items 18–20). The rating and scoring of the items remain unchanged (i.e., the same five‐point Likert scale with higher ratings indicating more negative attitudes). The factors are (1) role adequacy, (2) role support, (3) perceived competence and emotional response, and (4) job satisfaction.

### 2.5. Instruments of Measurement: Independent Variables

#### 2.5.1. Demographic Survey

At the beginning of the survey, participants reported demographic and professional information, including age, gender, unit of work (e.g., medical/surgical and critical care), nursing designation (RN or RPN), level of education, employment status, years of experience, prior education or training related to substance use/addiction (undergraduate and professional development), and self‐rated competence in caring for patients who use substances.

#### 2.5.2. Additional Variables

Four additional independent variables were examined: social desirability, missed nursing care, burnout, and ACEs.

##### 2.5.2.1. Balanced Inventory of Desirable Responding Questionnaire [34]

The 40‐item BIDR assesses social desirability bias across two scales: self‐deceptive enhancement (SDE; Items 1–20) and impression management (IM; Items 21–40). Responses are rated on a 7‐point Likert scale (0 = *not true* to 6 = *very true*). Items were dichotomized (responses 5–6 = 1, all others = 0) with select items reverse scored. Scale scores range from 0 to 20; higher scores indicate greater socially desirable responding. The BIDR has demonstrated good psychometric properties, with internal consistency coefficients of *α* = 0.83 and test–retest reliabilities of 0.69 and 0.65 for the scales [35].

##### 2.5.2.2. Workload Questionnaire [36]

The two‐part tool assesses missed nursing care: Part A (24 items; 5‐point Likert, 1 = *always missed* to 5 = *never missed*) and Part B (17 items; 4‐point Likert, 1 = *significant reason* to 4 = *not a reason*). Higher Part A scores indicate fewer missed care tasks; lower Part B scores indicate more significant barriers. The MISSCARE Questionnaire has demonstrated strong consistency and reliability (*α* = 0.64–0.86; [36]). Statistical analyses also have shown good model fit, with Bartlett’s test of sphericity (*p* < 0.001), a KMO value of 0.9, RMSEA = 0.054, CFI = 0.89, IFI = 0.9, and TLI = 0.85. The Pearson correlation coefficients are 0.87 for Part A and 0.86 for Part B [36]. Permission for usage was obtained from the creators.

##### 2.5.2.3. Burnout Questionnaire (Maslach Burnout Inventory Human Services Survey for Medical Personnel [37])

The 22‐item survey evaluates burnout across emotional exhaustion (EE; 9 items), depersonalization (DP; 5 items), and personal accomplishment (PA; 8 items). Items are rated 0–6 (0 = *never* to 6 = *every day*). Higher EE and DP scores and lower PA scores indicate greater burnout. The MBI‐HSS (MP) has demonstrated strong reliability and validity (*α* = 0.73–0.83; [38]). License to administer the MBI‐HSS (MP) was obtained on October 11, 2024, from Mind Garden Inc.

##### 2.5.2.4. ACEs Questionnaire [39]

The original 10‐item yes/no instrument assesses exposure to childhood adversity (score = sum of “yes” responses, 0–3 = low ACEs, and ≥ 4 = moderate/high ACEs). For this study, the questionnaire was adapted into a five‐item format to accommodate RNs and RPNs (e.g., time constraints [completing while on shift or on break]); work environment [busy, stressful, and multiple interruptions]; and the sensitive nature of the questions [emotional triggers while at work]) into a 5‐item modified version, with scores 0–2 = low and ≥ 3 = moderate/high ACEs. This adapted version has not been validated.

### 2.6. Rationale for Variable Selection

The independent variables included in this hierarchical regression model were selected based on prior empirical evidence and theoretical relevance to nurses’ attitudes. Professional and personal characteristics were entered in Block 1 as stable control variables, including demographics and social desirability (measured by the BIDR), to account for background influences and response biases. Variables previously identified as significant in the literature were prioritized although findings have been inconsistent. Block 2 comprised key psychosocial stressors, ACEs, MISSCARE, and MBI‐HSS [MP] measured using validated instruments, except for the modified ACEs questionnaire. These stressors were chosen because of their documented impact on nurses’ well‐being. The hierarchical regression approach enabled isolation of the unique effects of psychosocial stressors on attitudes after controlling for baseline characteristics.

### 2.7. Data Management and Analysis

Study data were gathered and stored using REDCap, an electronic data capture platform hosted by the principal investigator’s institution [40, 41]. To ensure anonymity and confidentiality, each participant in the current study was assigned a unique numeric identifier.

For the descriptive statistics, means and standard deviations were used for the quantitative variables, and frequencies and percentages were used for the categorical variables. Reliability of the AAPPQ, PC‐AAPPQ, DDPPQ, and PC‐DDPPQ scales, using the items and factor structure identified by Raynak and Colleagues [31], was examined using Cronbach’s alpha.

Given that participants may have completed either the regular or PC versions of the scales, we examined if both versions could be combined to increase the sample size available for the regression analyses; increasing the sample size would allow for the inclusion of more explanatory variables and adjustment of the models for more potential confounding variables. While wording differed between versions, item content, response scaling, and latent constructs were conceptually equivalent; therefore, we felt justified to examine if both versions could be combined. We conducted this analysis by comparing total and subscale means between versions using independent‐samples *t* tests, whereby the absence of statistically significant differences would suggest equivalency between versions. Unequal variances *t*‐tests were used when Levene’s test showed a violation of the equal variances’ assumption.

**FIGURE 1:**
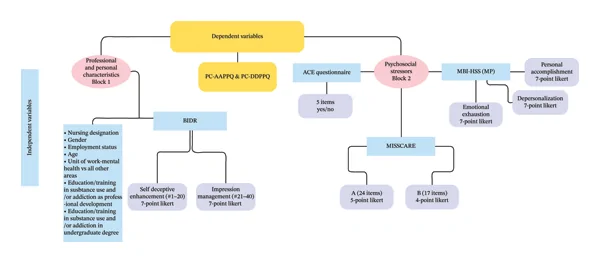
Visual depiction of hierarchical regression explanatory variables.

A hierarchical regression approach [42, 43] was used to examine associations between the dependent variables (combined AAPPQ and DDPPQ scales) and explanatory variables. Professional and personal characteristics’ explanatory variables were entered in Block 1. Psychosocial stressors were entered in Block 2. This hierarchical approach allowed the determination of the additional variance component explained by psychosocial stressors, above and beyond the contribution of variables entered in Block 1 (see Figure 1). To conserve degrees of freedom and to simplify the interpretation of the results, the variable representing hours of undergraduate education and professional development in substance use and/or addictions was collapsed from five categories (0, 1–4, 5–8, 9–12, and 13+ hours) to three (none, 1–8 h, and 9+ hours). In addition, the unit of work and the employment status of the nurse were combined into full time versus not and mental health inpatient unit versus not.

Regression coefficients (b) and associated *t*‐tests were examined to evaluate the direction and statistical significance of associations between explanatory variables and attitude scores. Negative regression coefficients would indicate associations with more positive attitudes, whereas positive coefficients would indicate associations with more negative attitudes. Overall model fit was assessed using the *F* test. Adjusted *R* ^2^ for each step and the change in the *F* ratio and *R* ^2^ between the two blocks were used to determine if the addition of variables in Block 2 improved the model. The level of significance 0.05 was used for all inferential analysis. All analyses were conducted using IBM SPSS v.30.

## 3. Results

This study examined the extent to which workload, burnout, ACEs, and social desirability were associated with nurses’ attitudes toward patients who use substances in hospital settings. Professional development in substance use and addiction demonstrated the most consistent association with more positive attitudes across both measures. Social desirability influenced responses, whereas burnout and workload demonstrated variable, scale‐dependent associations. ACEs were not significantly associated with attitudes.

### 3.1. Participant Characteristics

Four‐hundred and twelve (*N* = 412) nurses completed the surveys (response rate of 32%). The majority of the participants were women (89.9%) and RNs (83.3%) with a mean age of 36.76 (*SD* = 10.76). Over half of the participants had more than 10 years of experience in the profession (51.0%), and the majority of respondents were employed on a full‐time basis (57.0%). In terms of education, 62.9% held an undergraduate degree, 26.9% a diploma, and 9.2% graduate‐level education. Regarding substance use–related training, 44.7% reported 1–4 h of professional development, while 27.4% reported no training. Self‐rated competence in caring for patients who use substances was moderate (*M* = 6.39 and SD = 2.08) (Table 1).

**TABLE 1:** Participants’ characteristics.

Characteristics	Frequency (%), *M*, SD
Nursing designation	
RPN	65 (15.8%)
RN	343 (83.3%)
Missing data	4 (1%)
Age (*n* = 404)	*M* = 36.76, SD = 10.76, range 20–69
Highest level of education	
Diploma	111 (26.9%)
Undergraduate degree	259 (62.9%)
Graduate degree (i.e., Master’s)	38 (9.2%)
Missing	4 (1%)
Unit of work	
Critical care services	68 (16.6%)
Medical/surgical unit	120 (29.1%)
Mental health inpatient services	22 (5.3%)
Outpatient services	78 (18.9%)
Perioperative services	49 (11.9%)
Women and children services	49 (11.9%)
Other	15 (3.6%)
Missing	11 (2.7%)
Work status	
Full time	236 (57.3%)
Part time	118 (28.6%)
Casual	45 (10.9%)
Experience in role	
Up to 6 months	24 (5.8%)
> 6 months–2 yr	44 (10.7%)
> 2–5 yr	60 (14.6%)
> 5–10 yr	66 (16%)
> 10 yr	210 (51%)
Missing	8 (1.9%)
Experience in current unit	
Up to 6 months	43 (10.4%)
> 6 months–2 yr	80 (19.4%)
> 2–5 yr	92 (22.3%)
> 5–10 yr	83 (20.1%)
> 10 yr	104 (25.2%)
Missing	10 (2.4%)
Education/training received on substance use and/or addictions in nursing career	
None	113 (27.4%)
1–4 h	184 (44.7%)
5–8 h	56 (13.6%)
9–12 h	13 (3.2%)
> 13 or more hr	39 (9.5%)
Missing	7 (1.7%)
Hours of education/training received in undergraduate nursing program on substance use and/or addictions	
None	70 (17.0%)
1–4 h	175 (42.5%)
5–8 h	71 (17.2%)
8–12 h	48 (11.7%)
> 13 or more hr	35 (8.5%)
Missing	13 (3.2%)
Competence level (1–10, with 1 being *not confident* and 10 being *highly confident*) caring for PWUS in the hospital setting (*n* = 380)	*M* = 6.39, SD = 2.08

*Note: N* = 412.

### 3.2. Preliminary Analyses

Preliminary analyses were conducted to assess internal consistency and comparability between the original and person‐centered versions using the revised factor structures derived in the current study [31].

Internal consistency was acceptable for all scales (Cronbach’s *α* > 0.70), with one exception (PC‐DDPPQ perceived competence and emotional response, *α* = 0.66). Comparisons between versions demonstrated no meaningful differences in total or subscale scores, with one statistically significant but negligible difference. Given that both versions were scored using identical items and factor structures and demonstrated comparable reliability and score distributions, data were pooled for subsequent analyses (Table 2).

**TABLE 2:** AAPPQ and PC‐AAPPQ, DDPPQ, and PC‐DDPPQ scale descriptive characteristics.

Scale	No. of items	Cronbach’s alpha	*n*	*M* (SD)	Range	Comparison to PC versions *t* (*df*), *p*
*AAPPQ*	22	0.89	153	60.66 (10.71)	33–95	*t* (297.47) = 1.32, *p* = 0.188
Role adequacy	5	0.92	170	11.94 (3.53)	5–25	*t* (334) = 0.68, *p* = 0.498
Confidence, role clarity, and support	6	0.87	167	18.54 (4.64)	6–30	*t* (332) = 2.03, *p* = 0.043
Work satisfaction and motivation	6	0.81	163	15.21 (3.81)	6–25	*t* (322.61) = 0.38, *p* = 0.706
Self‐efficacy and emotional satisfaction	5	0.78	170	15.16 (2.93)	8–24	*t* (338) = 1.15, *p* = 0.252

*PC-AAPPQ*	22	0.86	154	59.16 (9.19)	36–97	
Role adequacy	5	0.91	166	11.68 (3.34)	5–25	
Confidence, role clarity, and support	6	0.84	167	17.57 (4.03)	6–30	
Work satisfaction and motivation	6	0.77	169	15.07 (3.39)	6–25	
Self‐efficacy and emotional satisfaction	5	0.77	170	14.81 (2.75)	5–24	

*DDPPQ*	16	0.91	144	44.97 (9.65)	16–74	*t* (299) = −0.02, *p* = 0.983
Role adequacy	7	0.92	151	19.72 (5.31)	7–35	*t* (309) = −0.53, *p* = 0.595
Role support	3	0.93	158	8.93 (2.90)	3–15	*t* (320) = 1.11, *p* = 0.269
Perceived competence and emotional response	3	0.77	159	7.53 (2.30)	3–15	*t* (321) = 1.10, *p* = 0.272
Job satisfaction	3	0.77	162	8.78 (2.08)	3–15	*t* (322) = −0.33, *p* = 0.585

*PC-DDPPQ*	16	0.87	157	44.99 (8.07)	25–70	
Role adequacy	7	0.90	160	20.03 (4.95)	7–35	
Role support	3	0.90	164	8.60 (2.48)	3–15	
Perceived competence and emotional response	3	0.66	164	7.27 (2.05)	3–13	
Job satisfaction	3	0.77	162	8.90 (1.99)	4–15	

### 3.3. Multivariable Regression Analyses

Multivariable regression analyses were conducted to examine factors associated with attitudes toward patients who use substances.

### 3.4. AAPPQ

For the AAPPQ, professional development in substance use and addiction was significantly associated with more positive attitudes across all scales. Age and gender demonstrated limited associations, with older nurses and female nurses reporting more negative attitudes on select domains. Social desirability influenced responses, with higher SDE scores associated with more positive attitudes across multiple scales, while IM showed limited and inconsistent associations.

Workload and burnout demonstrated mixed relationships. Higher DP was associated with more negative attitudes, whereas PA was associated with more positive attitudes. Emotional exhaustion showed limited but consistently positive associations in select domains. Missed care demonstrated selective associations, with Part A associated with more positive attitudes in specific domains and Part B associated with more negative attitudes in perceived competence (Table 3).

**TABLE 3:** Regression analysis for AAPPQ scales.

Explanatory variable		Role adequacy (*n* = 230)		Confidence, role clarity, and support (*n* = 234)		Work satisfaction and motivation (*n* = 236)		Self‐efficacy and emotional satisfaction (*n* = 241)	
		Block 2 (*b*, SE)	*t, p*	Block 2 (*b*, SE)	*t, p*	Block 2 (*b*, SE)	*t, p*	Block 2 (*b*, SE)	*t, p*
Constant		12.79 (2.23)	5.73	16.18 (2.85)	5.67	15.02 (2.16)	6.96	16.43 (1.91)	8.61
			< 0.001		< 0.001		< 0.001		< 0.001

Block 1	Age	0.03 (0.02)	1.07 0.284	0.08 (0.03)	2.67	0.01 (0.02)	0.37	−0.001 (0.02)	−0.04
					0.008		0.709		0.970
	Gender (female, compared with male)	0.69 (0.69)	1.00	1.64 (0.88)	1.87	1.54 (0.66)	2.33	−0.31 (0.59)	−0.53
			0.319		0.063		0.021		0.598
	Nursing designation (RPN, compared with RN)	0.21 (0.71)	0.30	−1.58 (0.90)	−1.76	−0.14 (0.67)	−0.21	−0.24 (0.60)	−0.41
			0.768		0.080		0.834		0.684
	Education/training in substance use and/or addiction as professional development (compared with no education)								
	1–8 h	−1.11 (0.54)	−2.05 0.041	−1.27 (0.68)	−1.87 0.063	−1.07 (0.52)	−2.07 0.040	−0.39 (0.46)	−0.85 0.398
	9+ hours	−2.34 (0.84)	−2.79 0.006	−2.38 (1.07)	−2.22 0.027	−1.59 (0.82)	−1.94 0.053	−1.99 (0.71)	−2.81 0.005
	Education/training in substance use and/or addiction in undergraduate education (compared with no education)								
	1–8 h	−0.15 (0.70)	−0.21 0.833	0.55 (0.87)	0.63 0.527	0.93 (0.67)	1.38 0.168	−0.43 (0.60)	−0.72 0.476
	9+ hours	−1.25 (0.82)	−1.52 0.130	−0.69 (1.04)	−0.66 0.509	−0.34 (0.80)	−0.42 0.675	−0.53 (0.71)	−0.75 0.455
	Mental health vs. all other units	−0.87 (0.93)	−0.93 0.353	−2.15 (1.16)	−1.86 0.064	−1.38 (0.88)	−1.56 0.119	−1.44 (0.76)	−1.88 0.061
	Full‐time employment vs. casual and part‐time employment	0.19 (0.49)	0.39 0.699	−0.39 (0.62)	−0.63 0.528	0.24 (0.47)	0.50 0.615	−0.08 (0.41)	−0.20 0.843
	BIDR SDE	−0.17 (0.08)	−2.11 0.036	−0.31 (0.10)	−3.01 0.003	−0.19 (0.08)	−2.37 0.019	0.03 (0.07)	0.47 0.642
	BIDR IM	0.05 (0.07)	0.76 0.448	0.22 (0.09)	2.36 0.019	−0.04 (0.07)	−0.57 0.567	−0.03 (0.06)	−0.53 0.595

Block 2	MISSCARE (A)	−0.01 (0.02)	−0.90 0.367	0.02 (0.02)	1.05 0.294	−0.02 (0.01)	−1.58 0.115	−0.03 (0.01)	−2.12 0.035
	MISSCARE (B)	0.05 (0.03)	1.96 0.051	−0.04 (0.03)	−1.05 0.294	0.02 (0.03)	0.81 0.418	0.04 (0.02)	1.68 0.095
	ACE Score	−0.18 (0.17)	−1.07	−0.08 (0.22)	−0.37	0.19 (0.17)	1.15	−0.18 (0.15)	−1.23
			0.286		0.714		0.251		0.219
	MBI‐HSS‐MP (EE)	−0.01 (0.23)	−0.05 0.959	0.18 (0.29)	0.63 0.530	0.34 (0.22)	1.55 0.122	−0.47 (0.19)	−2.47 0.014
	MBI‐HSS‐MP (DP)	0.02 (0.23)	0.07 0.943	−0.01 (0.29)	−0.05 0.961	0.65 (0.22)	2.92 0.004	0.95 (0.20)	4.90 < 0.001
	MBI‐HSS‐MP (PA)	−0.54 (0.23)	−2.14 0.034	−0.26 (0.32)	−0.80 0.424	−0.59 (0.25)	−2.42 0.017	−0.05 (0.22)	−0.23 0.816

Model fit Block 1		*F*(11,218) = 2.55, *p* = 0.005, *R* ^2^ = 11.4%		*F*(11,222) = 3.82, *p* ≤ 0.001, *R* ^2^ = 15.9%		*F*(11,224) = 3.88, *p* < 0.001, *R* ^2^ = 16.0%		*F*(11,229) = 2.24, *p* = 0.013, *R* ^2^ = 9.7%	

Model fit Block 2		*F*(17,212) = 2.28, *p* = 0.004, *R* ^2^ = 15.4%		*F*(17,216) = 2.63, *p* < 0.001 *R* ^2^ = 17.1%		*F*(17,218) = 5.12, *p* < 0.001, *R* ^2^ = 28.5%		*F*(17,223) = 3.45, *p* < 0.001, *R* ^2^ = 20.8%	

Change from Block 1 to Block 2		*F*(6212) = 1.68, *p* = 0.128, change in *R* ^2^ = 4.0%		*F*(6216) = 0.53, *p* = 0.783, change in *R* ^2^ = 1.2%		*F*(6218) = 6.36, *p* < 0.001, change in *R* ^2^ = 12.5%		*F*(6223) = 5.21, *p* < 0.001, change in *R* ^2^ = 11.1%	

### 3.5. DDPPQ

For the DDPPQ, professional development demonstrated strong and consistent associations with more positive attitudes across all domains. Working in a mental health inpatient unit was also associated with more positive attitudes. Demographic variables showed selective associations, with older nurses and female nurses reporting more negative attitudes in specific domains, while RPNs reported more positive attitudes than RNs on perceived competence and emotional response (Table 4).

**TABLE 4:** Regression analysis for DDPPQ scales.

**Explanatory variable**			**Role adequacy (*n* = 230)**		**Role support (*n* = 240)**		**Perceived competence and emotional response (*n* = 239)**				**Job satisfaction (*n* = 240)**		
			**Block 2 (b, *SE*)**	***t*, *p***	**Block 2 (b, SE)**	***t*, *p***	**Block 2 (b, SE)**		***t*, *p***		**Block 2 (b, SE)**	***t*, *p***	
	
Constant			18.50 (3.29)	5.62	7.12 (1.77)	4.02	6.08 (1.39)		4.39		9.58 (1.33)	7.20	
				< 0.001		< 0.001			*<* 0.001			*<*0.001	
Block 1	Age		0.07 (0.04)	1.83	0.06 (0.02)	2.91	0.01 (0.02)		0.58		0.002 (0.02)	0.13	
				0.068		0.004			0.566			0.900	
	Gender (female, compared with male)		2.27 (0.99)	2.29	0.19 (0.54)	0.35	−0.01 (0.43)		−0.03		0.21 (0.41)	0.52	
				0.023		0.726			0.975			0.607	
	Nursing designation (RPN, compared with RN)		−1.38 (1.02)	−1.35	−0.68 (0.55)	−1.22	−1.13 (0.43)		−2.62		−0.16 (0.43)	−0.37	
				0.178		0.223			0.009			0.715	
	Education/training in substance use and/or addiction as professional development (compared with no education)												
	1–8 h		−0.51 (0.79)	−0.64 0.521	−0.37 (0.42)	−0.88 0.379	−0.22 (0.33)		−0.66 0.509		−0.37 (0.32)	−1.16 0.249	
	9+ hours		−3.99 (1.21)	−3.30 0.001	−1.47 (0.65)	−2.25 0.026	−1.36 (0.51)		−2.67 0.008		−1.46 (0.49)	−2.96 0.003	
	Education/training in substance use and/or addiction in undergraduate education (compared with no education)												
	1–8 h		−0.47 (1.05)	−0.45 0.654	−0.62 (0.55)	−1.14 0.255	0.67 (0.43)		1.57 0.117		−0.02 (0.41)	−0.04 0.968	
	9+ hours		−2.12 (1.24)	−1.71 0.089	−0.83 (0.65)	−1.28 0.202	−0.08 (0.51)		−0.15 0.882		−0.40 (0.49)	−0.80 0.423	
	Mental health vs. all other units		−3.18 (1.32)	−2.41	−2.21 (0.71)	−3.13	−1.29 (0.55)		−2.33		−1.38 (0.53)	−2.59	
				0.017		0.002			0.021			0.010	
	Full‐time employment vs. casual and part‐time		0.67 (0.71)	0.93	−0.40 (0.38)	−1.05	−0.04 (0.30)		−0.12		0.45 (0.29)	1.54	
				0.352		0.297			0.902			0.124	
	BIDR SDE		−0.26 (0.12)	−2.17 *0.032*	−0.18 (0.06)	−2.78 *0.006*	−0.02 (0.05)		−0.47 0.063		−0.01 (0.05)	−0.18 0.857	
	BIDR IM		0.27 (0.11)	2.50 *0.013*	0.09 (0.06)	1.63 0.105	−0.05 (0.04)		−1.19 0.236		0.03 (0.04)	0.77 0.444	
Block 2	MISSCARE (A)		0.03 (0.02)	1.19 0.234	0.01 (0.01)	0.90 0.370	−0.01 (0.01)		−0.64 0.524		−0.02 (0.01)	−2.02 0.044	
	MISSCARE (B)		0.01 (0.04)	0.36 0.716	0.002 (0.02)	0.09 0.932	0.03 (0.02)		2.01 0.046		0.02 (0.02)	1.24 0.218	
	ACE Score		−0.01 (0.26)	−0.05	−0.02 (0.14)	−0.15	0.10 (0.11)		0.93		−0.002 (0.11)	−0.02	
				0.962		0.884			0.352			0.986	
	MBI‐HSS‐MP (EE)		−0.19 (0.34)	−0.55 0.584	0.03 (0.18)	0.16 0.877	0.04 (0.14)		0.27 0.785		−0.34 (0.13)	−2.56 0.011	
	MBI‐HSS‐MP (DP)		0.11 (0.34)	0.32 0.747	0.18 (0.18)	1.02 0.309	0.35 (0.14)		2.49 0.013		0.57 (0.14)	4.16 *<* 0.001	
	MBI‐HSS‐MP (PA)		−0.89 (0.38)	−2.35 *0.020*	−0.01 (0.20)	−0.06 0.949	−0.12 (0.16)		−0.77 0.440		−0.13 (0.15)	−0.84 0.405	
Model Fit Block 1			*F*(11,218) = 4.07, *p* < 0.001, *R* ^2^ = 17.0%		*F*(11,228) = 4.14, *p* < 0.001, *R* ^2^ = 16.7%		*F*(11,227) = 3.34, *p* < 0.001, *R* ^2^ = 14.3%				*F*(11,228) = 2.39, *p* = 0.008, *R* ^2^ = 10.3%		
Model Fit Block 2			*F*(17, 212) = 3.25, *p* < 0.001, *R* ^2^ = 20.7%		*F*(17, 222) = 2.91, *p* < 0.001, *R* ^2^ = 18.2%		*F*(17, 221) = 3.70, *p* < 0.001, *R* ^2^ = 22.1%				*F*(17, 222) = 2.94, *p* < 0.001, *R* ^2^ = 18.4%		
Change from Block 1 to Block 2			*F*(6212) = 1.63, *p* = 0.140, Change in *R* ^2^ = 3.7%		*F*(6222) = 0.72, *p* = 0.678, Change in *R* ^2^ = 1.6%		*F*(6221) = 3.73, *p* = 0.001, Change in *R* ^2^ = 7.9%				*F*(6222) = 3.66, *p* = 0.002, Change in *R* ^2^ = 8.1%		
	
**Explanatory variable (*b*, SE) *t, p***	**BIDR SDE**	**BIDR IM**	**MISSCA RE A**	**MISSCARE B**	**ACE score**	**MBI-HSS-MP (EE)**	**MBI-HSS-MP (DP)**	**MBI-HSS-MP (PA)**		**Model fit block 1**	**Model fit block 1**	**Change from block 1 to block 2**	
	
Role adequacy (*n* = 230)	−0.26 (0.12)	0.27 (0.11)	0.03 (0.02)	0.01 (0.04)	−0.01 (0.26)	−0.19 (0.34)	0.11 (0.34)	−0.89 (0.38)		*F*(11, 218) = 4.07, *p* < 0.001, *R* ^2^ = 17.0%	*F*(17, 212) = 3.25, *p* < 0.001, *R* ^2^ = 20.7%	*F*(6212) = 1.63, *p* = 0.140, Change in *R* ^2^ = 3.7%	
	−2.17, *0.032*	2.50, *0.013*	1.19, 0.234	0.36, 0.716	−0.05, 0.962	−0.55, 0.584	0.32 0.747	−2.35 *0.020*					
Role support (*n* = 240)	−0.26 (0.12)	0.27 (0.11)	0.01 (0.01)	0.002 (0.02)	−0.02 (0.14)	0.03 (0.18)	0.18 (0.18)	−0.01 (0.20)		*F*(11, 228) = 4.14, *p* < 0.001, *R* ^2^ = 16.7%	*F*(17, 222) = 2.91, *p* < 0.001, *R* ^2^ = 18.2%	*F*(6222)= 0.72, *p* = 0.678, Change in *R* ^2^ = 1.6%	
	−2.17, *0.032*	2.50, *0.013*	0.90, 0.370	0.09,0.932	−0.15, 0.884	0.16, 0.877	1.02, 0.309	−0.06 0.949					
Perceived competence and emotional response (*n* = 239)	−0.02 (0.05)	−0.05 (0.04)	−0.01 (0.01)	0.02 (0.02)	0.10 (0.11)	0.04 (0.14)	0.35 (0.14)	−0.12 (0.16)		*F*(11, 227) = 3.34, *p* < 0.001, *R* ^2^ = 14.3%	*F*(17, 221) = 3.70, *p* < 0.001, *R* ^2^ = 22.1%	*F*(6221) = 3.73, *p* = 0.001, Change in *R* ^2^ = 7.9%	
	−0.47, 0.063	−1.19, 0.236	−0.64, 0.524	2.01, 0.046	0.93, 0.352	0.27, 0.785	2.49 0.013	−0.77 0.440					
Job satisfaction (*n* = 240)	0.01 (0.05)	0.02 (0.04)	−0.02 (0.01)	0.03 (0.02)	−0.02 (0.11)	−0.34 (0.13)	0.57 (0.14)	−0.13 (0.15)		*F*(11, 228) = 2.39, *p* = 0.008, *R* ^2^ = 10.3%	*F*(17, 222) = 2.94, *p* < 0.001, *R* ^2^ = 18.4%	*F*(6222) = 3.66, *p* = 0.002, Change in *R* ^2^ = 8.1%	
	−0.18, 0.857	0.77, 0.444	−2.02, 0.044	1.24, 0.218	−0.02 0.986	−2.56 0.011	4.16 *<* 0.001	−0.84 0.405					

*Note:* Italics denote statistically significant values (*p* < 0.05).

Higher SDE scores were associated with more positive attitudes, while IM demonstrated limited negative associations. Burnout and workload again showed variable effects. Higher DP was associated with more negative attitudes, whereas emotional exhaustion showed limited but consistently positive associations in select domains. Missed care demonstrated selective associations, with Part A associated with more positive attitudes in specific domains and Part B associated with more negative attitudes in perceived competence. PA was associated with more positive attitudes in select domains.

Overall, modifiable factors, particularly professional development and workplace context, demonstrated the most consistent associations with attitudes, while individual characteristics and psychosocial stressors showed more variable effects.

## 4. Discussion

This study examined the extent to which workload, burnout, ACEs, and social desirability were associated with nurses’ attitudes toward patients who use substances in hospital settings. Hierarchical regression analyses identified multiple professional, personal, and psychosocial factors associated with attitudes; however, the direction and magnitude of associations varied by outcome scale and instrument (AAPPQ vs. DDPPQ), demonstrating the complex interplay of individual and system‐level factors shaping staff perceptions. Because all variables were measured contemporaneously, findings should be interpreted as statistical associations rather than causal or temporally predictive relationships.

At the individual level, age and gender emerged as relevant characteristics. Older nurses reported more negative attitudes on specific scales (e.g., confidence, role clarity and support [AAPPQ]; and role support [DDPPQ]), consistent with prior research showing that increased years of nursing experience can be associated with less favorable attitudes toward patients who use substances [10, 12, 14]. This finding may reflect cohort effects, habituation to challenging patient encounters, or professional socialization over time. Gender differences were also observed, with female nurses reporting more negative attitudes on certain scales (e.g., work satisfaction and motivation [AAPPQ] and role adequacy [DDPPQ]). For example, Molina‐Mula et al. [14], who examined emergency and mental health nurses’ attitudes toward individuals with alcohol use disorders, found that male nurses compared with female nurses exhibited a higher degree of rejection toward individuals with alcohol use disorder. Because these studies report similar associations, the result is not wholly unexpected; nonetheless, it suggests that education and support strategies may need to be tailored to mid‐ and late‐career nurses rather than assuming experience alone produces greater empathy or competence. For nursing leaders, these patterns highlight opportunities to develop targeted professional development and mentorship strategies that reflect workforce diversity in experience and generational training backgrounds.

Nursing designation emerged as a significant explanatory variable, as measured by the DDPPQ (e.g., perceived competence and emotional response), in which RPNs reported more positive attitudes on specific scales compared with RNs. This may reflect differences in educational preparation, scope of practice, typical clinical assignments, or workplace culture between designations. Because designation is a structural variable that organizations can identify easily, these results imply that tailoring professional development by designation (for example, role‐specific training or mentoring) may be worthwhile; however, the pattern is not yet well established in the literature and should be seen as a potentially novel and actionable observation warranting replication. Administrators may, therefore, consider designation‐specific training pathways or differentiated support models to optimize attitude change and clinical readiness across the nursing workforce.

In addition, nurses’ social desirability, as measured by the BIDR, was sometimes associated with attitudes toward patients who use substances across both measures. Social desirability likely produced conservative estimates of negative attitudes suggesting that true negative attitudes may be underestimated. This is expected in professions that emphasize nonjudgemental care. Methodologically, these results highlight the importance of measuring and accounting for social desirability in future analyses (and of triangulating self‐report with other data sources). From an administrative perspective, the presence of social desirability bias suggests that staff may not feel fully safe or supported expressing challenges in caring for patients who use substances, indicating a need for organizational cultures that support honest reflection and psychological safety.

Further analysis revealed that missed nursing care, as measured by the MISSCARE survey, and burnout, as assessed by the MBI‐HSS‐MP, exhibited variable associations with nurses’ attitudes toward patients who use substances across the AAPPQ and DDPPQ measures. For example, missed care (e.g., MISSCARE A) was related significantly to factors such as self‐efficacy, emotional satisfaction (AAPPQ), and job satisfaction (DDPPQ). Similarly, certain dimensions of burnout (e.g., MBI‐HSS‐MP) were significantly associated with work satisfaction (AAPPQ) as well as perceived competence and emotional response (DDPPQ). Findings were inconsistent across scales and measures, limiting definitive conclusions and suggesting context‐dependent effects. These findings should be interpreted with caution, and future researchers should aim to explore these variables further to determine if consistent patterns emerge. Nevertheless, the associations that did emerge reinforce that workplace conditions, staffing adequacy, workload, and burnout remain modifiable organizational factors that nursing administrators must address to foster more supportive practice environments and reduce the risk of negative attitudes.

ACEs were found to have no significant association with either measure. Previous research has shown a significant association between nurses’ attitudes and their personal connections to individuals who use substances (e.g., friends, family members, or coworkers), often resulting in more positive perspectives [7, 10, 11]. However, to our knowledge, this has not been previously examined as broader components of the ACEs questionnaire as potential explanatory variables for nurses’ attitudes toward patients who use substances. These findings suggest this may not be a priority area for future research. Although ACEs scores did not appear to influence the nurses’ attitudes toward patients who use substances, one variable that consistently showed an association across both attitude measures was education/training in substance use and/or addiction as professional development.

Education and professional development in substance use/addiction was consistently associated with more positive attitudes, and we observed a dose‐response pattern (1+ hour associated with improvement and 9+ hours associated with progressively more positive attitudes). This finding aligns with evidence that training improves provider attitudes [44–46] and with evidence that screening brief intervention referral to treatment (SBIRT) focused and empathy‐based interventions can shift role adequacy, legitimacy, motivation, and task‐specific self‐esteem [24, 46, 47]. A recent systematic review and meta‐analysis also supports the capacity of educational interventions to enhance evidence‐based knowledge and attitudes, particularly when multimodal delivery is used [48]. While causality cannot be inferred from this cross‐sectional analysis, the observed pattern strengthens the plausibility of a causal relationship; nonetheless, from a pragmatic standpoint, the association supports prioritizing ongoing professional development, especially multihour, multimodal programs that include SBIRT content and lived‐experience perspectives. For nursing administrators, these results provide clear direction: investment in substantive, well‐designed professional development initiatives is a high‐yield organizational strategy that can meaningfully shift staff attitudes toward patients who use substances. These findings position sustained, high‐quality professional development as the most actionable strategy for reducing stigma and improving care for patients who use substances in hospital settings.

Education received during undergraduate training was not associated with attitudes, whereas professional development education was (collinearity diagnostics confirmed these forms of education were analytically distinct). This discrepancy likely reflects differences in recency and applicability, with workplace‐based education being more timely and directly reinforced in practice. For organizations, this suggests that investment in continuing education targeted to practicing nurses will have greater immediate return in attitude change than relying on undergraduate preparation alone. However, it remains uncertain whether attitudinal improvements translate into measurable changes in patient care and patient experience. Most intervention studies to date emphasize provider self‐report outcomes, leaving a critical evidence gap on whether training leads to more compassionate, respectful, and equitable care as experienced by patients who use substances. Interpreting the literature and our findings together, measuring patient‐reported experiences and objective indicators of care quality should be a priority to determine the downstream impact of attitude change. These gaps emphasize a crucial leadership role for nursing administrators in supporting evaluation frameworks that link staff education to patient experience and quality outcomes.

Lastly, nurses working in mental health inpatient units demonstrated significantly more positive attitudes across all DDPPQ scales compared to nurses in other settings. This aligns with prior work showing more favorable attitudes among mental health nurses [14, 24] and may reflect greater exposure to, and specialized training in, recovery‐oriented and nonjudgemental approaches [49]. However, conflicting evidence and the possibility of self‐selection warrant cautious interpretation; instead, borrowing training content and cultural practices from mental health nursing may be more practical. This suggests an actionable opportunity for nursing leadership to integrate recovery‐oriented principles into broader hospital practice environments, potentially improving staff attitudes beyond mental health units.

These findings must also be interpreted within the broader context of stigma toward people who use substances, which remains pervasive in healthcare settings [50, 51]. Stigmatizing attitudes are well documented and negatively affect patient–provider interactions [50, 51], pain management [52], and care engagement [53]. While social desirability bias may mask the expression of negative attitudes, it does not eliminate their presence, highlighting the importance of interventions that move beyond knowledge acquisition to explicitly address stigma, bias, and structural factors within healthcare environments.

Within the Canadian healthcare context, where publicly funded hospital systems are experiencing sustained workforce pressures and increasing complexity of substance‐use presentations, these findings have particular relevance. Nursing shortages, high patient acuity, and variability in access to addiction‐related resources across regions may further shape attitudes and care practices. As such, organizational investments in education, harm reduction integration, and workforce supports are especially critical within Canadian acute care settings.

Future studies should (1) use designs that permit stronger causal inference (e.g., clustered trials or longitudinal intervention studies) to test whether educational dose produces sustained attitude and behavior change; (2) determine the optimal content and duration of professional development (that is, to confirm and specify the observed dose‐response threshold such as 9+ hours or another empirically derived cut‐point); (3) incorporate patient‐reported experience and objective care quality metrics to test whether attitudinal shifts translate to improved care; (4) employ mixed methods and measurement strategies that control for social desirability (for example, including BIDR scores in analytic models and using qualitative or observational triangulation); (5) further examine the context‐dependent roles of missed care and burnout, preferably with unit‐level and longitudinal data; and (6) investigate whether self‐selection or training differences explain the more positive attitudes among mental health nurses. Collectively, these steps will help move the field from documenting associations to identifying effective organizational and educational interventions to support nurse well‐being and equitable care.

### 4.1. Limitations

This study has several important limitations. First, data were self‐reported, which introduces the potential for social desirability bias and may not fully reflect nurses’ true attitudes or behaviors. Second, the observational, cross‐sectional design limits causal inference, and the instrument‐dependent patterning of effects means that some associations may be specific to the measures used rather than generalizable constructs. Third, a large number of variables and regression models were tested without correction for multiple comparisons, creating a risk of Type I error. Fourth, the limited gender diversity of the sample may reduce the generalizability of findings to more diverse nursing populations. Fifth, heterogeneity across clinical units and potential self‐selection into specialty areas may further constrain the applicability of results. Collectively, these limitations suggest that findings should be interpreted cautiously, and associations should be considered indicative rather than definitive. The last limitation relates to the pooling of the original and person‐centered versions of the AAPPQ and DDPPQ instruments using revised empirically derived factor structures. Although preliminary analyses supported comparable reliability and score distributions across versions, semantic differences in wording and the use of modified factor structures may limit comparability with prior studies using the original validated instruments.

## 5. Conclusion

Multiple factors including age, gender, designation, social desirability, missed care, burnout (variably), education/training, and work setting were associated with nurses’ attitudes toward patients who use substances, with education and mental health–based settings emerging as the most consistent correlates. For nursing administrators, these findings highlight that both workforce characteristics and modifiable organizational factors shape staff perceptions and the care environment. Practically, they support prioritizing thoughtfully designed professional development (multimodal, including SBIRT and lived‐experience content, of sufficient duration) as a scalable strategy to improve attitudes. These findings highlight sustained, high‐quality professional development as a key organizational strategy to reduce stigma and improve care for patients who use substances in hospital settings. They also emphasize creating practice environments reinforcing recovery‐oriented, nonjudgemental approaches, drawing lessons from mental health units. Further experimental and patient‐centered research is needed to confirm causal effects and determine whether improvements in attitudes translate into measurable gains in patient experience and care quality, an imperative for nursing leaders advancing equitable, stigma‐free care.

## Author Contributions

Andrea Raynak: conceptualization, data curation, formal analysis, investigation, methodology, project administration, resources, software, supervision, validation, visualization, writing–original draft, and writing–review and editing. Michel Bédard: supervision, validation, and writing–review and editing. Brianne Wood: supervision, validation, visualization, and writing–review and editing. Christopher Mushquash: supervision, validation, and writing–review and editing.

## Funding

Andrea Raynak’s work was supported by the Canadian Institutes of Health Research through the Doctoral Student Research Award, 186414.

## Disclosure

This manuscript is based on research conducted as part of the primary authors doctoral dissertation *(Examining nurses attitudes towards patients who use substances in the hospital setting)*, submitted to Lakehead University in 2025. Portions of the text, data, and analyses have been previously published in that thesis and are used here with permission. A full citation to the dissertation is included in the reference list.

## Conflicts of Interest

The authors declare no conflicts of interest.

## Data Availability

The data that support the findings of this study are available from the corresponding author upon reasonable request. Data are not publicly available due to institutional ethics and confidentiality restrictions.
